# Implementation and Conduct of Therapeutic Hypothermia for Perinatal Asphyxial Encephalopathy in the UK – Analysis of National Data

**DOI:** 10.1371/journal.pone.0038504

**Published:** 2012-06-13

**Authors:** Denis Azzopardi, Brenda Strohm, Louise Linsell, Anna Hobson, Edmund Juszczak, Jennifer J. Kurinczuk, Peter Brocklehurst, A. David Edwards, on behalf of the UK TOBY Cooling Register

**Affiliations:** 1 Centre for the Developing Brain, Imperial College London, London, United Kingdom; 2 National Perinatal Epidemiology Unit,University of Oxford, Oxford, United Kingdom; 3 Institute for Womens Health, University College London, London, United Kingdom; Erasmus University Rotterdam, Netherlands

## Abstract

**Background:**

Delay in implementing new treatments into clinical practice results in considerable health and economic opportunity costs. Data from the UK TOBY Cooling Register provides the opportunity to examine how one new effective therapy for newborn infants suspected of suffering asphyxial encephalopathy – therapeutic hypothermia- was implemented in the UK.

**Methodology/Principal Findings:**

We analysed returned data forms from inception of the Register in December 2006 to the end of July 2011. Data forms were received for 1384 (67%) of the 2069 infants registered. The monthly rate of notifications increased from median {IQR} 18 {15–31} to 33 {30–39} after the announcement of the results of the recent TOBY trial, and to 50 {36–55} after their publication. This rate further increased to 70 {64–83} following official endorsement of the therapy, and is now close to the expected numbers of eligible infants. Cooling was started at 3.3 {1.5–5.5} hours after birth and the time taken to achieve the target 33–34°C rectal temperature was 1 {0–3} hours. The rectal temperature was in the target range in 83% of measurements. From 2006 to 2011 there was evidence of extension of treatment to slightly less severely affected infants. 278 of 1362 (20%) infants died at 2.9 {1.4–4.1} days of age. The rates of death fell slightly over the period of the Register and, at two years of age cerebral palsy was diagnosed in 22% of infants; half of these were spastic bilateral. Factors independently associated with adverse outcome were clinical seizures prior to cooling (p<0.001) and severely abnormal amplitude integrated EEG (p<0.001).

**Conclusions/Significance:**

Therapeutic hypothermia was implemented appropriately within the UK, with significant benefit to patients and the health economy. This may be due in part to participation by neonatal units in clinical trials, the establishment of the national Register, and its endorsement by advisory bodies.

## Introduction

Delay in implementing new treatments into clinical practice once the evidence base has been established was identified as a ‘gap in translation’ by Sir David Cooksey in his review of UK Health Research, and results in considerable health and economic opportunity costs. [1] Data from the UK TOBY Cooling Register provides the opportunity to examine how one new effective therapy for newborn infants suspected of suffering asphyxial encephalopathy – therapeutic hypothermia- was implemented in the UK.

Extensive experimental and clinical research has been carried out into prolonged moderate hypothermia for perinatal asphyxial encephalopathy.[2]–[9] Synthesis of the results of randomised controlled trials show that the number needed to treat (NNT) with cooling to prevent one additional death or disabled survivor is 7, 95% confidence interval (CI) 4–14. [10], [11] Although reports from single institutions indicate that therapeutic hypothermia is increasingly used, so far there has been no report of national uptake of this therapy.

The UK TOBY Cooling Register was set up immediately following the conclusion of enrolment to the TOBY trial (ISRCTN8954757) a multicentre randomised controlled trial of whole body hypothermia for the treatment of perinatal asphyxial encephalopathy, supported by the Medical Research Council, UK, and predominantly carried out in the UK. [4] The objectives of the UK TOBY Cooling Register were to: provide guidance to clinicians considering the introduction of this therapy; audit the uptake and conduct of therapeutic hypothermia in the UK; and to undertake surveillance for adverse effects related to cooling. The UK TOBY Cooling Register became operational in December 2006 and since then all newborns treated with cooling in the UK have been eligible to be registered.

**Figure 1:**
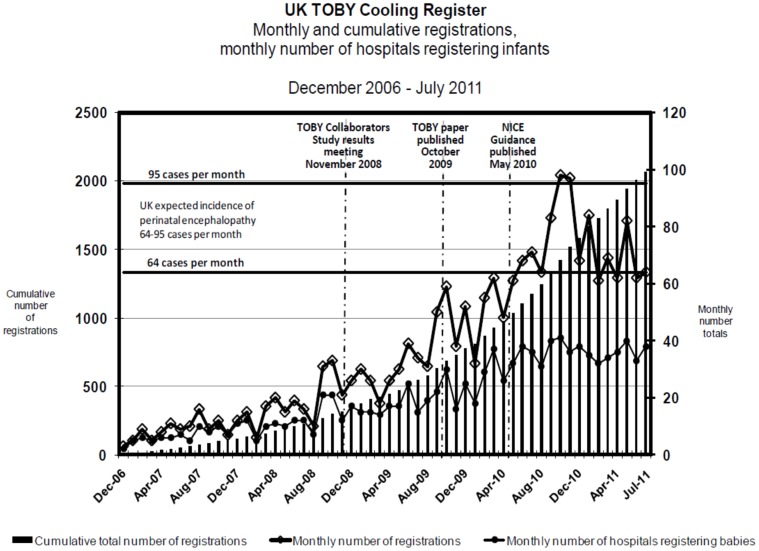
UK TOBY Cooling Register: number of registrations and cooling centres by month. The horizontal lines indicate the range of expected eligible cases in the UK.

When the results of the TOBY trial together with the other trials confirmed the therapeutic efficacy of prolonged moderate cooling, the TOBY study group planned the systematic implementation of cooling therapy throughout the UK. Register personnel produced a clinical protocol and handbook, and delivered a programme of presentations about therapeutic hypothermia to UK Neonatal Networks and at study days throughout the country. These supported the organisation of local services for the provision of hypothermia and the development of local clinical protocols. Subsequently, the introduction of cooling therapy into clinical practice and the reporting of cases to the Register were endorsed by the UK National Institute for Heath and Clinical Excellence (NICE) and the British Association of Perinatal Medicine. [12], [13].

**Figure 2:**
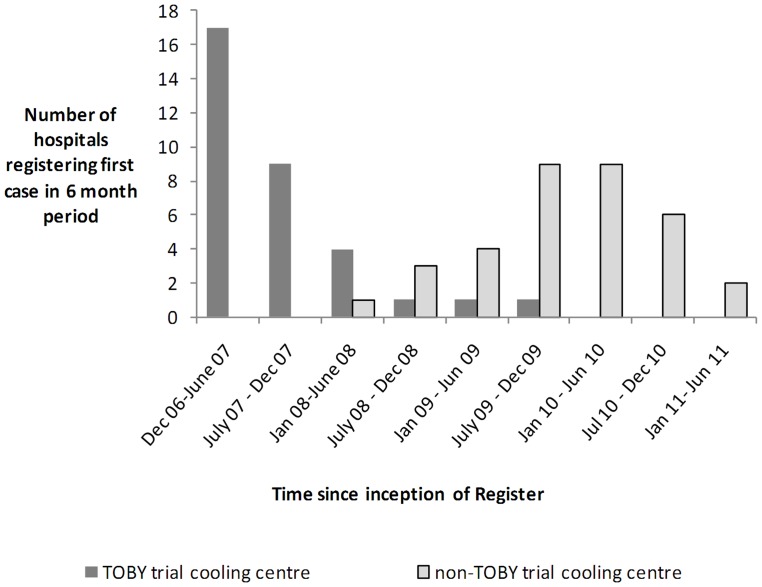
Number of hospitals registering first case in 6 month periods from inception of Register. Shaded bars indicate hospitals that participated in the TOBY trial and clear bars hospitals that did not participate in the TOBY trial.

**Figure 3:**
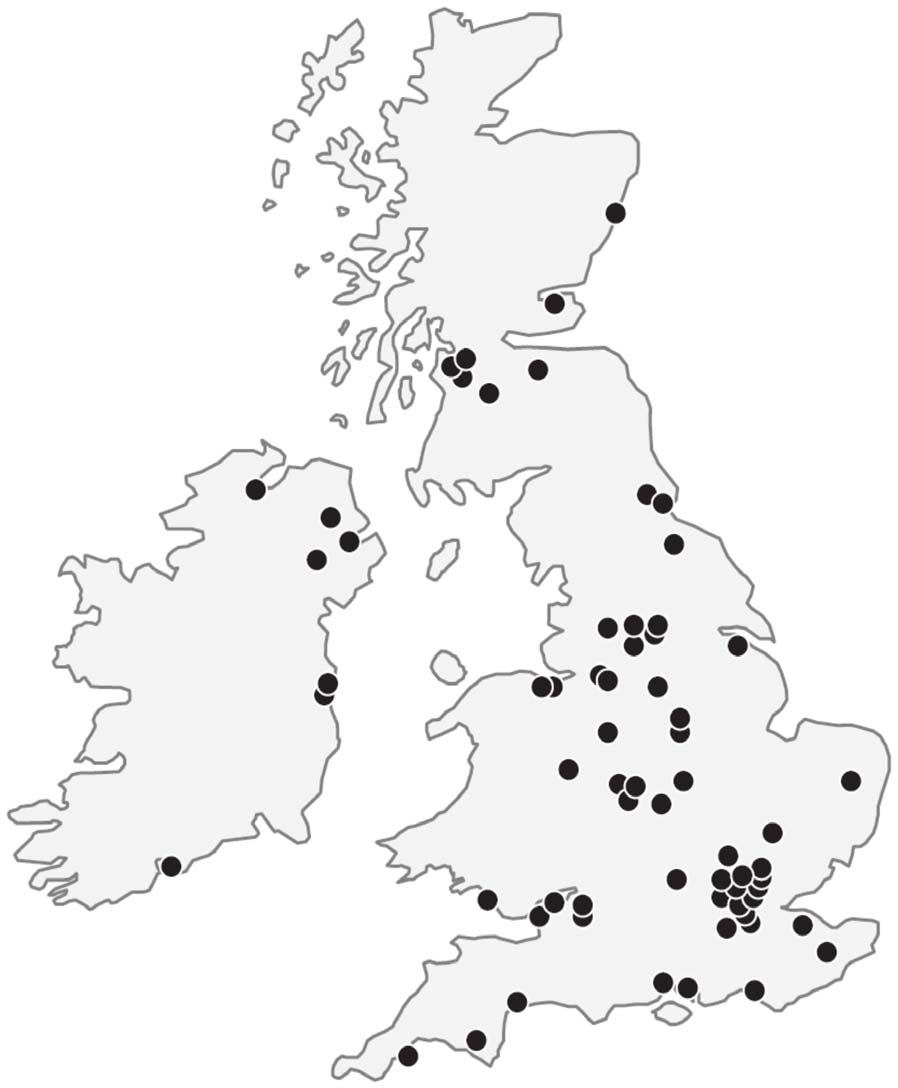
Distribution of cooling centres in the UK. Three centres from Eire contributed data from 35 cases and these are included in this report.

Based on previous UK population studies [14], [15], the prevalence of moderate to severe perinatal asphyxial encephalopathy is estimated to be 1–1.5/1000 births, and with a term birth rate of about 750,000 births [16], the number of infants that would be eligible for therapeutic hypothermia is estimated to be 750–1125 infants annually. This study aimed to determine whether there had been a ‘gap in translation’ in the implementation of therapeutic hypothermia, by: analysing the uptake of therapeutic hypothermia in the UK in relation to the developing evidence base; comparing the number of notifications with the expected health service provision needs; examining compliance with recruitment and treatment guidelines; and scrutinising the clinical characteristics and outcomes of treated infants relative to patients enrolled in the clinical trials.

**Figure 4:**
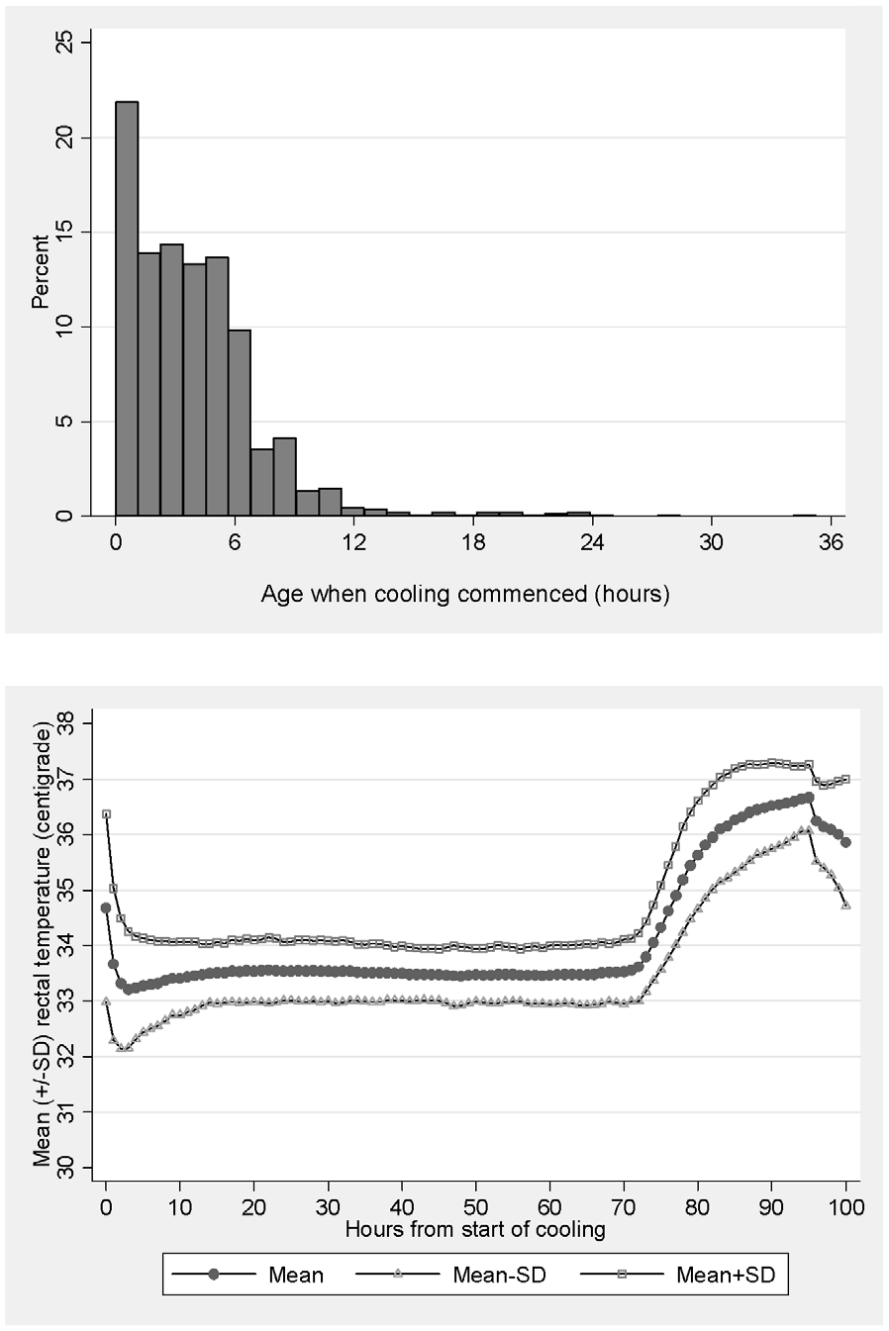
Frequencies of age at start of cooling in hours after birth.

**Figure 5:**
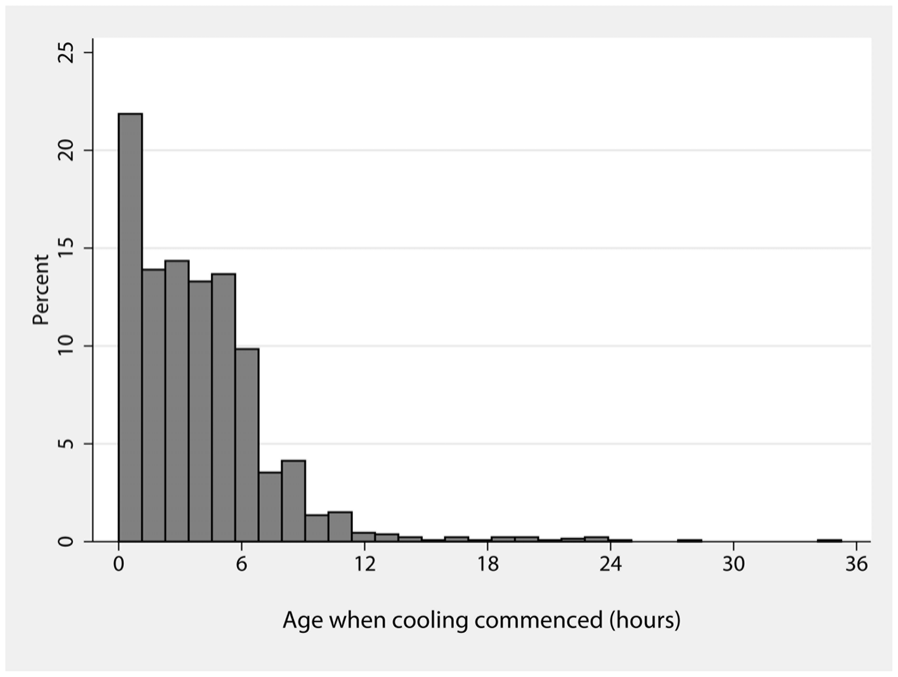
Mean hourly rectal temperature from the start of cooling to 96 hours of age.

## Methods

### The UK TOBY Cooling Register

The UK TOBY Cooling Register has been briefly described before [17], and the guidance and data forms are publicly available from the Register website: www.npeu.ox.ac.uk/tobyregister. The Register is located at the National Perinatal Epidemiology Unit (NPEU), Oxford, UK. It is managed by a co-ordinator (BS) and a clinical supervisor (DA), together with core personnel within the NPEU. Initial financial support for 12 months was provided by the UK Neonatal Task Force. The Register collects information about any newborn who receives treatment with cooling in the UK including Northern Ireland whether or not this was delivered according to the Register clinical protocol and guidelines, although there is no statutory obligation to register cases. All notified cases are allocated a unique patient identification number but no patient identifiers are collected, so consent from the parents for data collection is not required.

**Figure 6:**
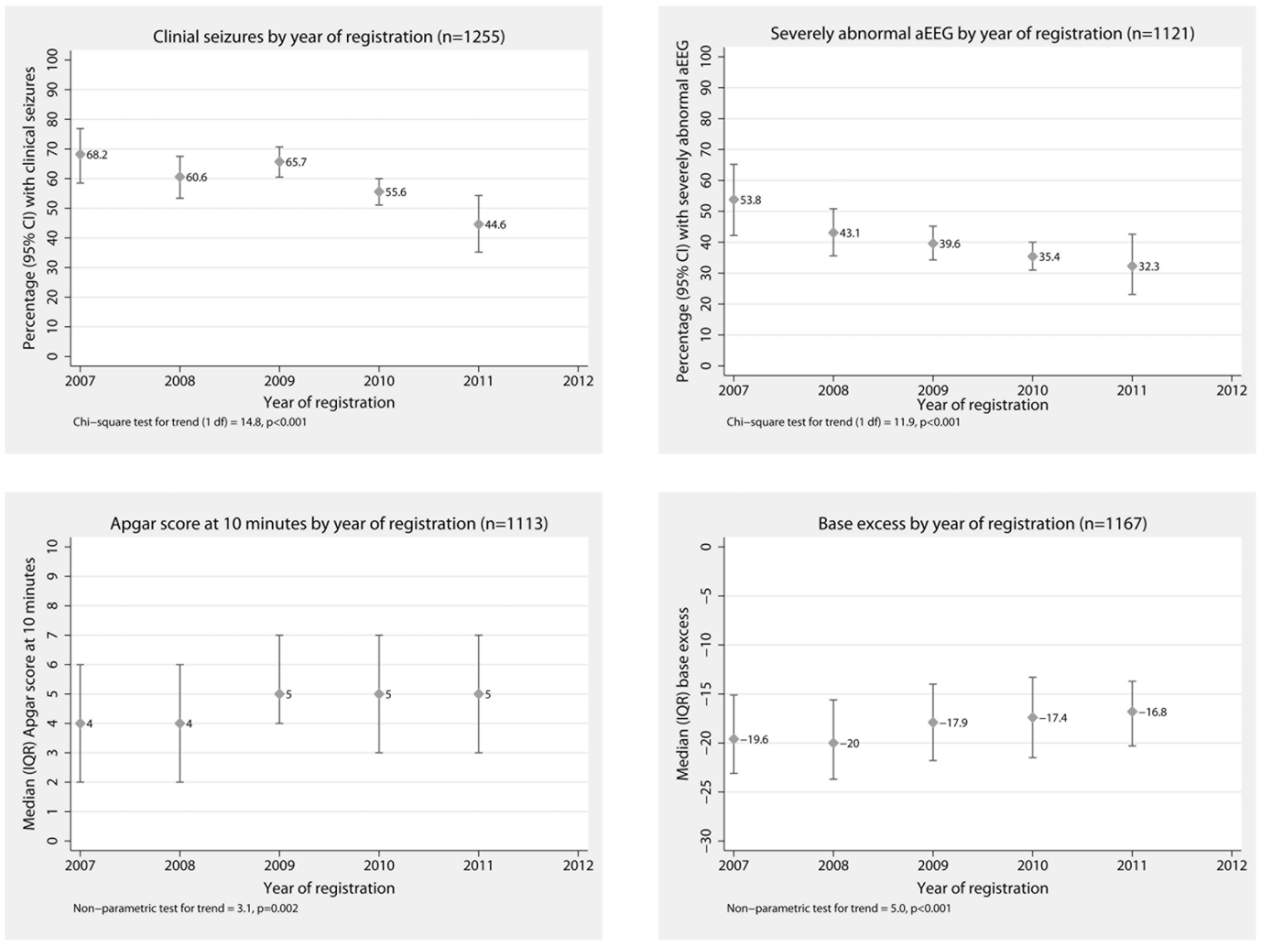
Clinical characteristics by year of registration.

The Register handbook gives guidance on which infants should be considered for treatment with cooling, with the aim to start cooling within six hours of birth. The indications are based on the criteria used in the TOBY trial, namely the presence in infants of at least 36 weeks gestation, of evidence of perinatal asphyxia together with signs of moderate or severe encephalopathy. Unlike in the TOBY trial, an abnormal amplitude integrated EEG (aEEG) recording is not a mandatory requirement, although clinicians are advised to perform aEEG recording whenever possible. This change from the TOBY trial was made primarily to avoid delay in initiating treatment with cooling when the aEEG is not available at the place of birth. The observation that patient characteristics and outcomes were similar amongst the randomised trials, yet only some included an abnormal aEEG as a trial entry criterion, suggests that it need not be a mandatory requirement.

**Table 1:** Key clinical characteristics of infants notified to the UK TOBY Cooling Register from December 2006 to July 2011.

Characteristic	n = 1384
Gestational age at birth, weeks (n = 1350)	
Median {IQR}	40 {38.4 to 41.1}
[Range]	[34.0 to 44.4]
Birth weight, grams (n = 1379)	
Median {IQR}	3340 {2900 to 3800}
[Range]	[1530 to 5830]
Age when cooling commenced, hours (n = 1331)	
Median {IQR}	3.3 {1.5 to 5.5}
[Range]	[0 to 35.3]
Cooling commenced after 12 hours of age, n (%) (n = 1331)	29 (2.2)
Cooled before gestational age of 36 weeks, n (%) (n = 1348)	38 (2.8)
Outcome at discharge, n (%) (n = 1362)	
Discharged home	912 (67)
Transferred to a different hospital	172 (13)
Died before discharge	278 (20)

Centres that introduce treatment with cooling are asked to nominate a lead contact person. An information pack about the Register and training material are provided and the Register co-ordinator provides specific guidance as required. Details of the neonatal centres notifying cases to the Register are available on the Register website. A monthly reminder note asking for a list of cases cooled in that month is sent to each registered cooling centre. Cases can be notified to the Register by telephone or email and this can be done retrospectively.

**Table 2:** Adverse events on days 1–4 after birth reported to the UK TOBY Cooling Register (Number of forms with completed data: 1384).

Event	Frequency (%)
Sepsis	232 (17)
Hypoglycaemia	344 (25)
Hypotension	557 (40)
Coagulopathy	435 (31)
Arrhythmia	118 (9)

Definitions:

Sepsis: Any evidence of infection requiring antibiotic therapy which is confirmed on culture.

Hypoglycaemia: Blood glucose below 2.6 mmol/litre.

Hypotension: Persistent mean blood pressure of <40 mmHg.

Coagulopathy: Any disorder requiring treatment in order to maintain or recover normal haemostasis.

Arrhythmia: Sinus bradycardia below 80 beats per minute and other arrhythmias identified on the electrocardiogram.

Clinical and treatment details are collected on a specific data record form that can be downloaded from the Register website. When the infant is approaching 2 years of age a request is sent to the notifying clinician asking for details of clinical outcomes at 2 years using a further downloadable record form; currently in the UK there is no statutory requirement for follow-up of infants who suffered neonatal encephalopathy or who received therapeutic hypothermia. Details sought are the findings on neurological examination, an assessment of neuromotor function using the Gross Motor Function Classification System (GMFCS) [18] and the Manual Abilities Classification System (MACS) [19] and the results of a neurodevelopmental assessment. The neurodevelopmental tests most commonly used in the UK are the Griffiths Mental Scales (Hogrefe Ltd, Oxford, England) and the Bayley Scales of Infant and Toddler Development (Pearson Assessment, London, England).

**Table 3:** Key clinical diagnoses reported to the UK Toby Cooling Register during hospital admission (Number of forms with completed data  = 1384).

Event	Frequency (%)
Meconium aspiration	144 (10)
Pulmonary haemorrhage	41 (3)
Pulmonary hypertension	99 (7)
Pulmonary airleak	63 (5)
Pneumonia	20 (1)
Late onset sepsis	30 (2)
Renal failure treated with dialysis	8 (0.6)
Necrotising enterocolitis	9 (0.7)
Major cerebral anomaly on cranial ultrasound	38 (3)

Definitions:

Meconium aspiration: The presence of meconium stained liquor at birth and severe respiratory distress within 1 hour of birth and compatible X-ray changes.

Pulmonary haemorrhage: Copious bloody secretions with clinical deterioration requiring change(s) in ventilatory management.

Pulmonary hypertension: Severe hypoxaemia disproportionate to the severity of lung disease and evidence of a right to left shunt.

Pulmonary airleak: Any radiologically confirmed airleak serious enough to affect management (including pneumothorax, pulmonary interstitial emphysema, pneumopericardium, pneumoperitoneum and pneumomediastinum).

Late onset sepsis: Any evidence of infection after 72 hours from birth requiring antibiotic therapy which is confirmed on culture.

Necrotising enterocolitis: Infants with abdominal distension, gastric aspirate and/or blood in stools together with abdominal X-ray showing bowel oedema, pneumatosis or pneumoperitoneum, i.e. Bell’s staging 2 or 3.

Major cerebral anomaly on cranial ultrasound: Including evidence of parenchymal haemorrhage as determined by ultrasound, ventricular dilatation (defined as >97th centile for gestational age) or the presence of porencephalic cysts or cystic leukomalacia.

A chart of each infant’s temperature record is sent back to notifying clinicians if requested, following receipt of the data record forms and summary information about their cases is available to all registered cooling centres. Notification, use of Register material, feedback service and online and telephone support are provided freely to all clinicians and the entire Register website is publicly accessible.

**Table 4:** Two year outcomes of infants notified to the UK TOBY Cooling Register (Number forms with completed data  = 275).

Outcome	n	(%)
Gross Motor Function Classification System (GMFCS), (n = 65)*		
Level 1–2	38	(58)
Level 3–5	27	(42)
Manual Ability Classification System (MACS), (n = 65)*		
Level 1–2	36	(55)
Level 3–5	29	(45)
Cerebral palsy (n = 251)	56	(22)
Bilateral 2 limb	4	(2)
Bilateral 4 limb	29	(12)
Hemiplegic left	6	(2)
Hemiplegic right	3	(1)
Dystonic	5	(2)
Choreoathetoid	1	(–)
Ataxic	3	(1)
Non-classifiable	5	(2)
Occipital head circumference (cm), (n = 196)		
Mean (SD)	48	(2.5 )

* 15 children with GMFCS/MACS levels 1–2 were recorded as not having cerebral palsy and 5 children recorded as having cerebral palsy were not classified using these scales.

The numbers of hospitals registering their first case is shown in Figure 2. Only those hospitals that participated in the TOBY trial registered cases in the first 18 months after inception of the Register; other hospitals began registering cases after the TOBY trial results became available. Figure 3 shows the distribution of cooling centres in the UK. Three centres from Eire contributed data from 35 cases. Of 74 UK neonatal units that notified the Register of the intention to provide therapeutic hypothermia, 54 were classed Level 3 (highest level of care) units, 11 were classed Level 2 units; the level of care was indeterminate in the remaining 9 units. The median {IQR} number of infants cooled in each unit were 29 {20–52} in Level 3 neonatal units and 3 {0–11} in the Level 2 neonatal units.

### Statistical Methods

We analysed returned data forms from inception of the Register in December 2006 to the end of July 2011. We report the individual and cumulative number of notifications and treatment centres contributing to the Register. Numbers (with percentages) are presented for binary and categorical variables, and means (with standard deviations) or medians (with lower and upper quartiles ({IQR}) for continuous variables as appropriate.

We used poisson regression to examine the difference in the rate of monthly registrations 6 months before and the 6 months after the results of the TOBY trial became available, their publication and the publication of the NICE guidelines. We analysed the temperature profiles and clinical details of notified cases, including severity of encephalopathy assessed by a modification of the Sarnat score [3] or by the Thompson score [20], and outcome at discharge and at 2 years of age. We examined trends in clinical characteristics over time using the Chi-squared test for trend for categorical variables and the non parametric test for trend for ordinal and continuous variables. [21]

We investigated risk factors for adverse outcome at 2 years for those with available outcome data. Adverse outcome at 2 years was defined as death, cerebral palsy and/or a GMFCS or MACS score >2. First we examined the association of the following risk factors with adverse outcome in a bivariate analysis using Fisher’s exact test: clinical seizures prior to cooling, the aEEG, Apgar score at 10 minutes, cord gas pH, base excess and Thompson encephalopathy score prior to cooling. Significant factors in the bivariate analysis (p value<0.05) were included in a multivariate logistic regression analysis.

Two-sided 5% significance tests and 95% confidence intervals are reported throughout. Stata/SE version 11.1 was used for all data analysis.

### Ethics and Consent

The UK Central Office for Research Ethics Committees advised that Research Ethics Committee review was not required as the Register was considered a service evaluation. Subsequently the UK National Research Service also advised that since no patient identifiers are collected there is no statutory requirement for consent from the parents for data collection and consent was not sought. The Register guidelines advise clinicians to discuss offering treatment with cooling with the parents and to follow local practice as to whether formal consent for treatment should be sought, but we did not collect this information from registering centres.

## Results

2069 notifications were received by the end of July 2011. Figure 1 shows the monthly and cumulative registrations and contributing hospitals since inception of the Register. The monthly rate of notifications increased from median {IQR} 18 {15–31} in the 6 month period before the announcement of the results of the TOBY trial in November 2008, 33 {30–39} in the 6 months before the publication of the TOBY trial results in October 2009 and 50 {36–55} in the following 6 months; the rate of registrations increased further in the 6 months following publication of the NICE endorsement of therapeutic hypothermia in May 2010 when a median {IQR} 70 {64–83} infants monthly were notified to the Register. Since then, the monthly rate of notifications has remained fairly steady at a median {IQR} of 68 {62–82}.

Clinical characteristics after birth were consistent with moderate/severe asphyxial injury in most cases (Figure 6). Of 513 infants with completed Thompson encephalopathy scores prior to initiation of cooling, with lower scores indicating milder encephalopathy, 91 infants (18%) scored 0–5, 265 infants (51%) scored 6–12, and 157 infants (31%) scored >12.

### Clinical Characteristics

At the time of data analysis in July 2011, data forms had been received for 1384 (67%) of the 2069 infants registered and the key clinical characteristics of these infants are shown in Table 1.

In 1331 cases with data available, cooling was started at a median {IQR} of 3.3 {1.5–5.5} hours after birth and the time to achieve the target 33–34°C rectal temperature was a median {IQR} of 1 {0–3} hours. The distribution of the age in hours at start of cooling is shown in Figure 4. For 29/1331 (2.2%) infants, cooling was commenced more than 12 hours after birth. The method of cooling was whole body cooling in all but 3 cases; servo controlled equipment was used in half the cases during the last 12 months. The mean hourly rectal temperature from the start of cooling is shown in Figure 5. The rectal temperature was in the target range in 83% of 79,584 measurements recorded during the maintenance phase of cooling from 8 to 72 hours. Excessive cooling to below 33°C occurred in 887/1368 (65%) infants at some point, but this was mostly in the 32–33°C range. The rectal temperature was below 31°C at some point in 61/1368 (4%) of infants.

The proportion of infants with suspected clinical seizures before cooling started decreased over the time period of the Register (Figure 6). Similarly the proportion of infants with a severely abnormal aEEG grade reported prior to cooling decreased over time. There was also an improvement in Apgar scores at 10 minutes and first blood base excess from 2007 to 2011. In the last 12 month period, the proportion of infants with seizures before cooling started was 49.2% (95% CI 43.5% to 54.9%); the proportion with a severely abnormal aEEG was 34.0% (95% CI 28.5% to 39.9%); the median {IQR} Apgar scores at 10 minutes were 5 {3 to 7}; and the base excess median {IQR} −17.1 {−21.0 to −13.3}.

Among 1121 babies with data recorded over the first four days, the proportion of infants with suspected clinical or aEEG seizures progressively fell from 62% at 24 hours of age to 13% on the 4th day after birth. There was a similar progressive improvement in the encephalopathy score during the same period. Among 1384 infants, respiratory support with mechanical ventilation or continuous positive airways pressure was required in 1144 (82%) on day 1, 842 (61%) on day 2, 673 (49%) on day 3 and 466 (34%) on day 4.

Thirty eight of 1350 (2.8%) cases were born before 36 weeks gestation; 4 were born at 34 weeks and the others were born at 35 weeks. The median Apgar scores were 1, 4 and 5 at 1, 5 and 10 minutes and the median pH and base excess were 6.82 and −19.1 mmol/L in the cord blood or first blood sample after birth. Suspected seizures occurred in 17/33 (52%) before cooling was started and placental abruption was reported in 18/38 (47%) cases.

### Adverse Events (Table 2)

Potential adverse events during the first four days after birth that were included in the Register data forms are shown in table 2, and the frequency of key clinical conditions during the hospital admission period is shown in table 3.

We have recently reported the occurrence of subcutaneous fat necrosis in approximately 1% of registered infants. [22] Three cases of inadvertent excessive cooling due to malfunction of equipment caused by a broken temperature probe were notified and these incidents were reported to the Medical Devices Agency (UK). Other unusual events included the occurrence of one case of vitreous haemorrhage, with full recovery; hearing loss in 2/7 children from one centre where aminoglycosides were routinely administered; a case of collapse associated with severe unexplained hypertension after rewarming, again with full recovery; and reports of severe bradycardia following lidocaine infusion, which prompted reinforcement of the guidance to reduce infusion rates and duration during cooling.

### Outcomes at Discharge

Of 1362 infants with known outcomes during the first hospital admission, 278 (20%) died before discharge at a median {IQR} of 2.9 {1.4 to 4.1} days of age and 172 (13%) were transferred to a different hospital (Table 1). The mortality rate decreased from 24/109 (22%) in 2007 to 17/117 (15%) in 2011, Chi-squared for trend P = 0.08, but there may be a time lag in the notification of deaths in the current year. Full enteral feeding was achieved by 777/1046 (74%) of survivors before discharge at a median {IQR} age of 8 {6 to 12} days; this did not alter significantly during the study period. Among infants born before 36 weeks gestation, 12 (32%) infants died at a median {IQR} of 2.5 {1.7 to 4.2} days of age, and 17/24 (71%) of survivors achieved full enteral feeding before discharge at a median {IQR} of 14 {8 to 17} days.

### Outcomes at 2 Years

Outcome data at 2 years of age were received from 275/574 (48%) of requests (Table 4). There were no significant differences in the clinical characteristics, aEEG prior to cooling, and the age in hours after birth when cooling was started between the infants with or without 2 year outcome data available. Cerebral palsy was clinically diagnosed in 56/251 (22%) infants and half of these were spastic bilateral involving 4 limbs. Levels 3–5 (indicating severe neuromotor disability) were recorded in 27/51 (53%) infants with cerebral palsy assessed with the GMFCS and in 29/51 (57%) infants assessed with the MACS.

Formal neurodevelopmental assessment was carried out in 135/249 (54%) of cases, but a developmental quotient was only obtained in 34 cases; in the other children it could not be obtained or the forms were incomplete, so it was not possible to explore factors associated with neurodevelopmental outcome. A further 8/275 (3%) died following discharge from hospital and before 2 years.

Risk factors significantly associated with adverse outcome at 2 years in the bivariate analyses were: clinical seizures prior to cooling, severely abnormal aEEG, Apgar score at 10 minutes, base excess and Thompson encephalopathy score prior to cooling. The Thompson encephalopathy score was not included in the multivariate model because the score was only available for 70 babies with 2 year outcome data. In the multivariate analysis of adverse outcome (n = 149), the factors that were independently associated with adverse outcome were clinical seizures prior to cooling (p<0.001) and severely abnormal aEEG (p<0.001).

## Discussion

Intervention with moderate cooling for neural rescue in newborns with hypoxic-ischaemic brain injury is the culmination of a series of research studies spanning decades. These proved the potential for neural rescue following perinatal asphyxia [23]; consistently showed benefit from post-insult cooling in appropriate experimental models [24], [25]; examined safety and feasibility in preliminary clinical studies [26], [27]; and confirmed efficacy by synthesis of the results of several well conducted randomised clinical trials in newborns [10].

The UK TOBY Cooling Register data indicate that there has been timely, systematic implementation of therapeutic hypothermia in the UK to a standard protocol, so successfully bridging the ‘gap in translation’ from research to clinical use. [1] From the inception of the Register there was a steady rise in the number of notifications that intensified once the results of the TOBY trial were announced, and increased further following official endorsement of this therapy.

Because of a lack of systematic recording, the incidence of neonatal encephalopathy in the UK is uncertain as are the outcomes following encephalopathy. The current rate of notifications to the UK TOBY Cooling Register is very close to our estimate that 750–1125 infants annually would be eligible for therapeutic hypothermia based on previous UK population studies and the term birth rate. We suggest that the rapid uptake and now consistently high rate of provision of therapeutic hypothermia was aided by two factors. First, several UK neonatal centres participated in the TOBY trial and so were ready to implement therapeutic hypothermia into clinical practice; this highlights the value to a healthcare system of active participation in clinical trials. Second, the guidance and the clinical feedback provided nationally by the Register, which demonstrates the value to patients and the UK Health Economy of continued research and development funding following the completion of pragmatic trials.

In a meta analysis of the clinical trials of cooling the mortality rate in the cooled groups was 25% and the rate of disabling cerebral palsy was 21% in survivors, which are very similar to our data from infants reported to the national Register of therapeutic hypothermia in the UK. [10] Since treatment with cooling for 72 hours cooling is cost saving by 18 months of age, it is likely to have important benefits to the health economy in countries where the intervention is applied widely. [28] From the number of infants treated with cooling since the inception of the Register and the NNT of 7 infants to prevent one additional disabled survivor reported from the clinical trials, using current lifetime costs for each child with cerebral palsy (about £750,000) [29] and the economic benefit of additional healthy lives (about £800,000) [30], the total benefit to the UK economy as a result of the implementation of therapeutic hypothermia is likely to be in excess of £125 million so far.

In the absence of any specific therapy for a devastating condition, and following encouraging if not definitive results of two previous randomised controlled trials [31], when recruitment for the TOBY trial was complete many clinicians wished to offer therapeutic hypothermia on compassionate grounds before the procedure had been assessed by the regulatory authorities. Therapeutic hypothermia was first notified to NICE before the results of the TOBY trial were available, so the review process was postponed until publication of the trial results. The UK TOBY Cooling Register was ideally placed to support those clinicians that wished to offer therapeutic hypothermia whilst also providing specialist advice to the NICE review process so that a co-ordinated approach to implementing this therapy was achieved.

An initial concern expressed by regulatory authorities and specialist bodies is that therapeutic hypothermia may be applied outside the criteria used in the clinical trials- that is a ‘therapeutic drift’ to treatment with cooling in less severely affected newborns or in conditions other than suspected perinatal asphyxia encephalopathy. [12], [13] Indeed, the clinical condition at birth improved, and the percentage of infants with clinical seizures before cooling reduced over the period of the Register (Figure 6); however, the clinical characteristics of registrants in the last 12 month period of the Register remained consistent with treatment guidelines.

It is to be expected that outside a clinical trial, clinicians will explore therapeutic options more widely on compassionate grounds. For any therapy, clinicians must consider how applicable the supporting evidence is to the individual case, the risk of side effects and the availability of other treatments; it is not sufficient to rely solely on the eligibility criteria of clinical trials, and clinicians may opt to treat patients that differ in some respects from those entered in the clinical trials. [32], [33] The UK TOBY Cooling Register guidelines provide advice on the use of cooling outside clinical trial criteria such as after 6 hours from birth or in infants born before 36 weeks gestation or after presumed postnatal hypoxic-ischaemic acute events. The gestational age was the same as in the TOBY trial ( = >36 weeks gestation) in all but 3% of registrants, and the lowest gestation reported was 34 weeks. The mortality rate in the infants born before 36 weeks gestation was greater than in the whole group, 12/38 (30%) compared with 278/1362 (20%); this observation may be due to selection bias but it highlights the need to critically assess the extension of therapeutic hypothermia to premature infants. Overall these data suggest that only minor ‘therapeutic drift’ occurred over the duration of the Register but continued surveillance is important.

It has been suggested that the severity of encephalopathy assessed clinically or by aEEG is less predictive of subsequent outcome when infants are treated with hypothermia. [34]–[36] However, in another study of infants treated with hypothermia, severe encephalopathy and an abnormal aEEG were related to outcome at 18 months of age. [37] Register data suggest that in addition to the severity of encephalopathy and the aEEG, the occurrence of clinical seizures prior to cooling is also a risk factor for adverse outcome at 2 years of age. These clinical features may identify a group of infants who might benefit by earlier institution of therapeutic hypothermia or who may be candidates for studies of additional neuroprotective therapies. Experimental studies support the concept that the ‘therapeutic window’ is shorter and hypothermia needs to be started earlier in more severely affected infants. [38].

Adverse events, such as biochemical and metabolic abnormalities and coagulopathy were frequent during the first four days after birth but it is likely that these were due to the severity of perinatal asphyxia rather than treatment with cooling. Culture proven sepsis was reported in 17% of cases in the first 72 hours following birth, and occurred in just 30/1384 (2%) after the end of cooling, suggesting that sepsis was unlikely to be related to treatment with cooling. Pneumonia has been associated with therapeutic hypothermia in adult studies but this complication was reported in less than 2% of cases notified to the Register. Apart from subcutaneous fat necrosis, which was reported in approximately 1% of cases, other adverse conditions occurred with a similar frequency to that reported in the clinical trials.

There are a number of limitations to the analysis of data from registers, such as: difficulty in ascertaining uptake of registration especially when the incidence of the disorder is not known; reporting bias; and incomplete or erroneous data. Therefore, caution is necessary when interpreting the data especially when comparing outcomes with those reported in clinical trials or with historical data. However, registers are ideal for monitoring practices and patient outcomes outside the narrow context of clinical trials. The UK TOBY Cooling Register was set up specifically to monitor the introduction of therapeutic hypothermia in the UK in infants with perinatal asphyxial encephalopathy. Registration of cases is entirely voluntary; we aimed to encourage participation in the Register by providing clinical guidance including individual advice if required, specific data forms that could be used as part of the clinical record and clinician feedback. The number of notifications during the last 12 months of the Register was close to the estimated number of infants eligible for treatment, which suggests that our data provide a representative account of the use of this therapy in the UK.

The Register data show that in the UK most infants are treated safely and appropriately, according to treatment criteria, within 6 hours of birth, with good temperature control and few complications related to cooling. The rates of death and severe disability are similar to those reported in the randomised controlled trials of hypothermia. These data indicate that therapeutic hypothermia was implemented appropriately within the UK, with significant benefit to patients and the UK health economy. This may be due in part to participation by UK neonatal units in clinical trials, the establishment of the national Register, and its endorsement by national advisory bodies.
